# Dual Mechanisms of Action: Anti-Candida and Anti-Inflammatory Potential of *Lactobacillus* Fermentation Broth in Treating Vulvovaginal Candidiasis

**DOI:** 10.3390/jof11010018

**Published:** 2024-12-30

**Authors:** Huann-Cheng Horng, Jin-Wei Xu, Yi-Shan Kuo, Yu-Sin Chen, Yu-Hsuan Chiu, Kuan-Hao Tsui, Yu-Tang Tung

**Affiliations:** 1 Department of Obstetrics and Gynecology, Taipei Veterans General Hospital, Taipei 112, Taiwan; 2 Department of Obstetrics and Gynecology, National Yang Ming Chiao Tung University, Taipei City 112304, Taiwan; 3 Institute of Clinical Medicine, National Yang Ming Chiao Tung University, Taipei City 112304, Taiwan; 4 Faculty of Medicine, College of Medicine, Fu-Jen Catholic University, New Taipei City 24205, Taiwan; 5 Department of Forestry, National Chung Hsing University, Taichung 402, Taiwan; 6 Graduate Institute of Biotechnology, National Chung Hsing University, Taichung 402, Taiwan; 7 School of Medicine, College of Medicine, National Yang Ming Chiao Tung University, Taipei City 112304, Taiwan; 8 Department of Obstetrics and Gynaecology, Kaohsiung Veterans General Hospital, Kaohsiung 813, Taiwan; 9 Advanced Plant and Food Crop Biotechnology Center, National Chung Hsing University, Taichung 402, Taiwan; 10 Cell Physiology and Molecular Image Research Center, Wan Fang Hospital, Taipei Medical University, Taipei 110, Taiwan

**Keywords:** vulvovaginal candidiasis (VVC), *Candida albicans*, *Lactobacillus*, inflammation, fermentation broth

## Abstract

Vulvovaginal candidiasis (VVC), a condition predominantly caused by *Candida albicans*, affects millions of women worldwide, prompting the need for alternative treatments due to the side effects and increasing resistance associated with conventional imidazole antifungals. This study investigated VAGINNE^®^, a novel fermentation broth derived from *Lactobacillus* species, as a potential VVC treatment. Using a BALB/c mouse model of *C. albicans* infection, we evaluated VAGINNE^®^’s effects on vaginal microbiome composition, inflammatory markers, and tissue integrity. Our findings revealed that VAGINNE^®^ treatment enhanced the growth of beneficial *Lactobacillus* species while suppressing *C. albicans* proliferation, leading to a more balanced vaginal microbiome. Additionally, VAGINNE^®^ significantly reduced pro-inflammatory cytokines (IL-17A, IL-22, IL-23) in vaginal tissues and systemic inflammatory markers (IL-6, IL-1β) in plasma. Histological analysis showed minimal fungal invasion and preserved vaginal epithelial integrity in VAGINNE^®^-treated mice compared to untreated controls. These results suggest that VAGINNE^®^ could serve as an effective anti-Candida and anti-inflammatory agent for managing VVC, offering a promising alternative to traditional antifungal treatments. By promoting a healthy vaginal microbiome, reducing inflammation, and maintaining tissue health, this probiotic-based approach presents a novel strategy for addressing VVC, particularly in cases of drug resistance or adverse reactions to standard therapies. This study underscores the potential of microbiome-modulating strategies in managing vaginal infections, paving the way for more targeted and side-effect-free VVC treatments.

## 1. Introduction

Vulvovaginal Candidiasis (VVC) is a prevalent mucosal infection primarily caused by the *Candida* species, affecting an estimated 75% of women of reproductive age at least once in their lifetime. This condition is characterized by genital discomfort, including itching, a burning sensation, and dyspareunia, often accompanied by a distinctive vaginal discharge. Notably, 5–8% of women experience recurrent VVC (RVVC), presenting a significant clinical challenge [1,2]. *Candida albicans*, a common commensal organism in the human microbiome, can transition to a pathogenic state under certain conditions. This transition is facilitated by various factors, including vaginal dysbiosis, immune evasion mechanisms, and the expression of virulence factors such as hyphal growth, biofilm formation, and the production of proteolytic enzymes and Candidalysin [3]. The disruption of the vaginal microbiota, particularly the reduction in *Lactobacillus* populations, has been implicated in enhancing *Candida*’s ability to invade vaginal epithelial cells [4]. The pathogenesis of VVC involves the activation of the NLRP3 inflammasome in epithelial cells by *Candida*’s pseudohyphae and hyphae, leading to a pronounced inflammatory response [5,6,7]. Current therapeutic approaches primarily rely on topical and/or oral antifungal agents, with azole antifungals being the predominant treatment modality [7]. These agents function by inhibiting ergosterol production and interfering with fungal cell wall synthesis [8,9]. However, the management of VVC faces significant challenges [10]. The fungistatic nature of azole antifungals and the inherent resilience of fungal organisms contribute to the recurrence of infections. Moreover, the widespread and repeated use of antifungal agents for both prophylaxis and treatment has led to the emergence of resistant *Candida* strains [11,12,13]. This growing concern about antifungal resistance has stimulated research interest in exploring alternative therapeutic options, particularly those derived from natural sources.

The vaginal microbiome is predominantly composed of *Lactobacillus* species, with *L. iners*, *L. crispatus*, *L. jensenii*, and *L. gasseri* being the most prevalent [14,15]. These *Lactobacillus* species employ various mechanisms to maintain vaginal health, including biofilm formation, adherence to epithelial surfaces, production of antimicrobial substances, nutrient competition, and immunomodulation [16,17,18,19]. Recent studies have demonstrated the inhibitory effects of *Lactobacillus*-derived supernatants on *C. albicans* growth, filamentation, adhesion, and biofilm formation, suggesting the presence of secretory antimicrobial compounds beyond the well-known hydrogen peroxide and organic acids [16,17]. The host immune response to *C. albicans* infections involves both innate and adaptive mechanisms. While early research emphasized the importance of Th1 responses, characterized by the production of INF-γ, TNF-β, IL-6, and IL-2 [16,18,19,20], recent findings have highlighted the critical role of Th17-mediated immunity in protecting against mucosal and disseminated fungal infections [21]. Th17 cells, induced by IL-1β, IL-6, and TGF-β, and further promoted by IL-23, produce IL-17 and IL-22 [22]. These cytokines are crucial in orchestrating inflammation, neutrophil recruitment, and tissue repair during *C. albicans* infections [21].

The metabolic products of *Lactobacillus* species, particularly lactic acid, play a crucial role in maintaining an acidic vaginal pH, which is inhospitable to many pathogens. Recent research has revealed that these metabolites not only directly inhibit *C. albicans* growth but also modulate its virulence factor expression. For instance, short-chain fatty acids produced by *lactobacilli* have been shown to downregulate genes associated with hyphal formation and biofilm production in *C. albicans*, potentially reducing its pathogenicity [16,18,19]. Furthermore, the immunomodulatory effects of *Lactobacillus* species extend beyond their direct antimicrobial actions. Studies have demonstrated that certain *Lactobacillus* strains can enhance the production of antimicrobial peptides by vaginal epithelial cells and modulate the recruitment and activation of immune cells [16]. This multifaceted approach to host defense underscores the potential of probiotic-based therapies in managing VVC. The emergence of antifungal resistance in *Candida* species, particularly non-albicans *Candida*, has further complicated the treatment landscape for VVC [11,12,13]. While *C. albicans* remains the predominant causative agent, the increasing prevalence of species such as *C. glabrata* and *C. krusei*, which often exhibit reduced susceptibility to azole antifungals, highlights the need for alternative therapeutic strategies [8,9]. Recent advances in genomics and metabolomics have provided new insights into the molecular mechanisms underlying the antagonistic relationship between the *Lactobacillus* and *Candida* species. Transcriptomic analyses have revealed complex regulatory networks in both organisms that respond to each other’s presence, suggesting potential targets for therapeutic intervention [17]. The concept of using *Lactobacillus*-derived fermentation broths as a treatment for VVC represents a shift towards harnessing the natural defense mechanisms of the vaginal microbiome. This approach aligns with the growing interest in microbiome-based therapies across various medical fields. However, the translation of these findings into clinical applications presents several challenges, including the standardization of probiotic formulations, the optimization of delivery methods, and ensuring stability and efficacy of the fermentation products [6,7]. Despite these advancements, there remains a significant gap in the literature regarding in vivo assessments of the anti-*Candida* effects of *Lactobacillus*-derived fermentation broths. Furthermore, the practical implications of scaling up the production of supernatants from *L. crispatus*, *L. gasseri*, and *L. jensenii* for VVC treatment require thorough investigation.

To address these knowledge gaps and explore the therapeutic potential of *Lactobacillus*-derived products, this study employed a VVC model induced by a *C. albicans* infection in BALB/c mice. The objective was to assess the efficacy of a fermentation broth derived from *L. crispatus*, *L. gasseri*, and *L. jensenii* in mitigating VVC symptoms and modulating the host immune response. This approach aims to provide valuable insights into the development of novel, probiotic-based strategies for managing VVC, potentially offering an alternative to conventional antifungal treatments [1,2,3,4,5].

## 2. Materials and Methods

### 2.1. Animal Model

Female BALB/c mice, aged 7 weeks and weighing between 18 and 21 g, were sourced from BioLASCO Taiwan Co., Ltd. (Taipei, Taiwan). The mice were housed in individually ventilated cages under standard laboratory conditions, which included a controlled temperature of 22 ± 2 °C, humidity of 60 ± 5%, and a 12-h light/dark cycle. They had free access to food and water throughout the study. After a one-week acclimatization period to ensure environmental adjustment, the animals were used for experimentation. All experimental protocols were approved by the Institutional Animal Care and Use Committee of National Chung Hsing University (IACUC NO. 110-096R). These conditions and protocols ensured optimal health and welfare of the mice throughout the study, minimizing stress factors that could influence experimental outcomes.

### 2.2. Preparation of VAGINNE^®^

VAGINNE^®^, derived from the fermentation broth of *Lactobacillus crispatus*, *L. gasseri*, and *L. jensenii*, is produced through a carefully controlled process. The bacterial strains are incubated in bovine milk at 30–40 °C for 16–18 h. Chitin, a fermentation promoter, is added to the culture to enhance the growth of *Lactobacillus* species. The pH is maintained between 3.5 and 4.5. Following incubation, the culture is centrifuged at 3000 rpm for 10 min at 3–10 °C, and the supernatant is filtered using a 0.22 µm filter. The resulting supernatant, containing bacteriocins, oligosaccharides, and lactic acid, is collected. VAGINNE^®^ was supplied by the JoyCom Group (Kaohsiung, Taiwan). The final product is rich in bioactive compounds that support microbial balance and inhibit pathogenic organisms.

### 2.3. Animal Model and Treatment Protocol

**Figure 1:**
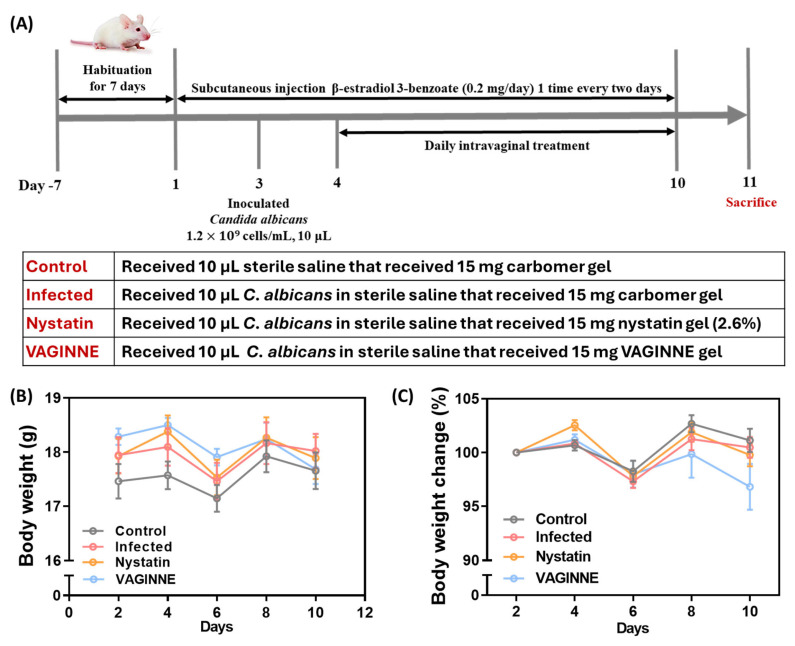
Experimental design and physiological monitoring of vaginal candidiasis model. The timeline (**A**) illustrates the study progression, beginning with β-estradiol 3-benzoate priming via subcutaneous route, followed by intravaginal *C. albicans* introduction. Therapeutic interventions were administered from Days 4 to 10, with groups receiving either carbomer gel (Infected controls), Nystatin, or VAGINNE^®^, while uninfected controls received vehicle gel only. Biological specimens (vaginal lavage, tissue, and blood) were collected at study termination (Day 11). Graphs demonstrate the temporal body mass measurements (**B**) and weight variations (**C**) throughout the infection course.

A vaginal candidiasis mouse model was established following a modified protocol from previous studies [23]. The experimental timeline is illustrated in Figure 1A. Female BALB/c mice were subcutaneously injected with 0.2 mg of β-estradiol 3-benzoate in 100 μL of sesame oil 48 h prior to vaginal infection (Day 1). To maintain pseudo-estrus, β-estradiol 3-benzoate was administered every other day throughout the study period (Day 1–10). *Candida albicans* blastoconidia were cultured in Sabouraud Dextrose Broth (Becton Dickinson and Company, Difco^TM^; Franklin Lakes, NJ, USA) for 24 h at 25 °C. The cultures were washed thrice with sterile saline, and the concentration was adjusted to 1.2 × 10^9^ cells/mL using a hemocytometer. On Day 3, mice were intravaginally inoculated with 10 μL of the blastoconidial suspension, except for the uninfected control group, which received sterile saline.

The mice were randomly assigned to four groups (*n* = 6 per group):

1. Control: Uninfected, received carbomer gel;
2. Infected: Received *C. albicans* and carbomer gel;
3. Nystatin: Received *C. albicans* and 2.6% nystatin gel (positive control);
4. VAGINNE^®^: Received *C. albicans* and VAGINNE^®^ gel.

Treatment administration was conducted as follow: Each cohort of mice received their designated intravaginal interventions on a daily basis during Days 4–10. Throughout the experimental period, both nutritional intake and weight measurements were documented to generate developmental trajectories.

Specimen collection was administered in the following way: Following the completion of the treatment, terminal procedures were conducted on Day 11. The animals were placed under deep anesthesia using isoflurane (4–5%, Minrad, NY, USA). A visual examination of vaginal secretions was conducted in accordance with established methodologies. For microbiological assessment, the vaginal environment was irrigated using a measured volume (100 μL) of sterile physiological saline. The resulting lavage specimens were retained for subsequent quantification of bacterial populations and inflammatory mediators. Vaginal discharge was visually assessed following the protocol described by Guo et al. [24].

Blood analysis took place as follows: Retro-orbital collection technique was employed to obtain blood specimens. An aliquot (300 μL) was dispatched to Accuspeedy Medical Laboratory (Tainan, Taiwan) for comprehensive leukocyte profiling. To isolate plasma components for immunological analysis, the remaining blood fraction underwent high-speed centrifugation (3000× *g*) for 15 min under refrigerated conditions (4 °C).

All samples for cytokine measurements were stored at −80 °C until further analysis. This comprehensive approach allowed for the evaluation of both local and systemic responses to *C. albicans* infection and subsequent treatments.

### 2.4. Colony Counts of C. albicans and Lactobacillus *spp.*

The method, based on Sun et al. [23], was modified for this study. Vaginal lavage fluids were serially diluted in 0.9% saline and plated onto a Candida Plus medium (50.9 g in 1 L of purified water) and an MRS medium (55 g in 1 L of purified water) to culture *Candida albicans* and *Lactobacillus* species, respectively. The plates were incubated at 30 °C for 48 h. After incubation, colony-forming units (CFUs) were counted to quantify *C. albicans* and *Lactobacillus* populations, which provided insights into the microbial dynamics within the vaginal environment during infection and treatment.

### 2.5. Tissue Histopathology

Preparation of vaginal specimens occurred in the following manner: Following euthanasia, vaginal tissue samples were harvested and preserved in neutral buffered formalin (10%) for a 24-h fixation period. The preserved tissues underwent sequential dehydration through ascending ethanol concentrations (starting at 70% and progressing through 85% and 95%, to absolute ethanol). Following dehydration, the specimens were processed for paraffin embedding and cut into 4-μm sections.

Microscopic analysis took place as follows: Standard H&E staining procedures were performed to visualize tissue architecture and pathological alterations. Microscopic examination was conducted using an Olympus BX51 platform (Tokyo, Japan). A qualified veterinary pathologist evaluated the specimens for infection-related changes, employing established assessment parameters adapted from previous studies by Shackelford and colleagues [25], along with Munyaka’s research team [26]. Detailed scoring metrics are provided in the (Supplementary Documentation Tables S1 and S2).

### 2.6. Enzyme-Linked Immunosorbent Assay (ELISA)

Vaginal lavage fluid was collected by gently introducing sterile PBS or saline into the vaginal canal with a sterile pipette and then aspirating it back, repeating the process 2–3 times to obtain sufficient fluid. The collected fluid was transferred to a microcentrifuge tube, centrifuged to remove cellular debris, and stored at −80 °C until ELISA analysis. Systemic cytokines, including IL-6, IL-1β, and IFN-γ, were quantified in plasma, while local immune responses were assessed by measuring IL-17A, IL-22, and IL-23 in vaginal tissue. Additionally, IL-17A, an indicator of Th17 cell response, was analyzed in vaginal lavage fluid to evaluate localized immune dynamics. Cytokine quantification was performed using ELISA kits (BioLegend, San Diego, CA, USA) following the manufacturer’s protocols, specifically targeting IL-17A (432504), IL-22 (436304), IL-23 (433704), IL-6 (431304), IL-1β (432604), and IFN-γ (430804), to provide insights into systemic and localized immune activities.

### 2.7. Statistical Analysis

All statistical analyses were performed using GraphPad Prism 6.01 (San Diego, CA, USA). The results are presented as the mean ± standard error of the mean (SEM). Statistical analysis was performed using a one-tailed Mann–Whitney U test. * *p* < 0.05 and ** *p* < 0.01 indicate comparisons with the control group, while ^#^ *p* < 0.05 and ^##^ *p* < 0.01 indicate comparisons with the Infected group.

## 3. Results

### 3.1. Effect of Fermentation Broth on Vaginal Discharge and Inflammation in C. albicans-Infected Mice

We conducted a comprehensive evaluation of the antifungal properties of VAGINNE^®^, a fermentation broth derived from *Lactobacillus crispatus*, *Lactobacillus gasseri*, and *Lactobacillus jensenii*, using a well-established experimental murine model of vaginal candidiasis (VVC) (Figure 1A). In this model, female BALB/c mice were induced into a pseudoestrus state and subsequently inoculated intravaginally with *Candida albicans* to simulate an infection. Following inoculation, the mice were treated with either VAGINNE^®^ or Nystatin, the latter serving as a positive control for antifungal efficacy. Mice were sacrificed on day 11 post-inoculation to facilitate a thorough assessment of treatment outcomes, including both histological and microbiological evaluations. Throughout the study, we monitored the body weight of the mice to evaluate any adverse effects related to the treatments. Our results indicated no significant differences in body weight (Figure 1B) or weight change (Figure 1C) across the four experimental groups: Control, Infected, Nystatin, and VAGINNE^®^. These findings suggest that VAGINNE^®^ is well-tolerated in this model, setting the stage for further investigations into its efficacy in managing VVC.

**Figure 2:**
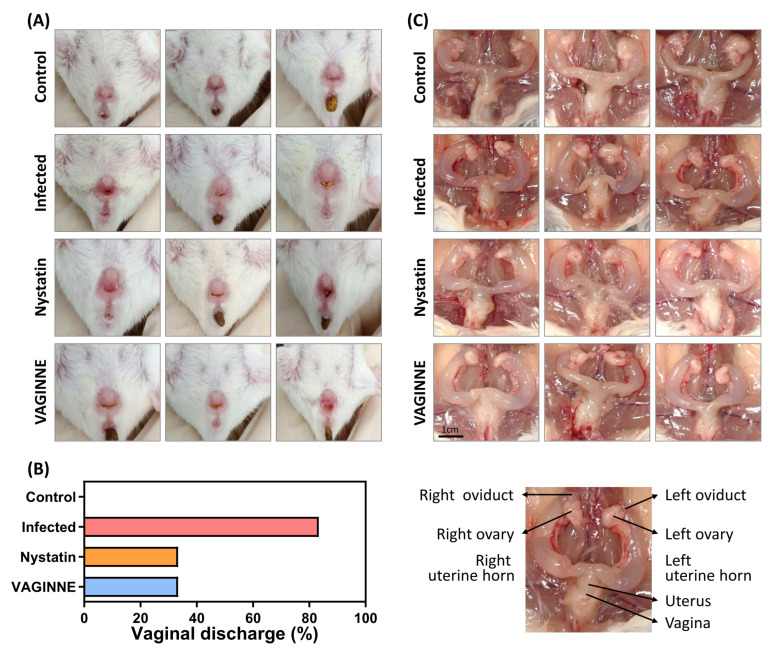
Therapeutic impact of VAGINNE^®^ administration in a murine *C. albicans* infection model. Representative images showing alterations in (**A**) vaginal structural characteristics, (**B**) secretory discharge patterns, and (**C**) uterine architecture across treatment groups. Experimental cohorts comprised untreated controls, infection controls receiving carbomer gel, standard antifungal treatment (nystatin gel), and VAGINNE^®^ intervention groups.

As depicted in Figure 2A,B, a striking 83.3% of the mice in the Infected group (five out of six) demonstrated significant vaginal discharge, a clear indication of the severity of the candidiasis. In stark contrast, those treated with VAGINNE^®^ experienced a substantial reduction in discharge, with only two out of six mice exhibiting similar symptoms, showcasing an efficacy on par with the standard treatment, Nystatin. Moreover, VAGINNE^®^ treatment led to noticeable improvements in the external appearance of the vaginal tissue, characterized by diminished inflammation. As shown in Figure 2C, the treated mice exhibited a healthier, less inflamed appearance compared to the infected controls. Additionally, fur quality served as an indirect indicator of overall health; while the Infected group displayed dull and uneven fur coloration, indicating systemic effects of the infection, the Control, Nystatin, and VAGINNE^®^-treated groups presented smoother and more vibrant coats. This observation not only underscores the therapeutic potential of VAGINNE^®^ in alleviating local symptoms but also suggests systemic benefits that contribute to overall well-being.

### 3.2. Impact of Fermentation Broth on C. albicans and Lactobacillus spp. Colony Counts in Vaginally Infected Mice

**Figure 3:**
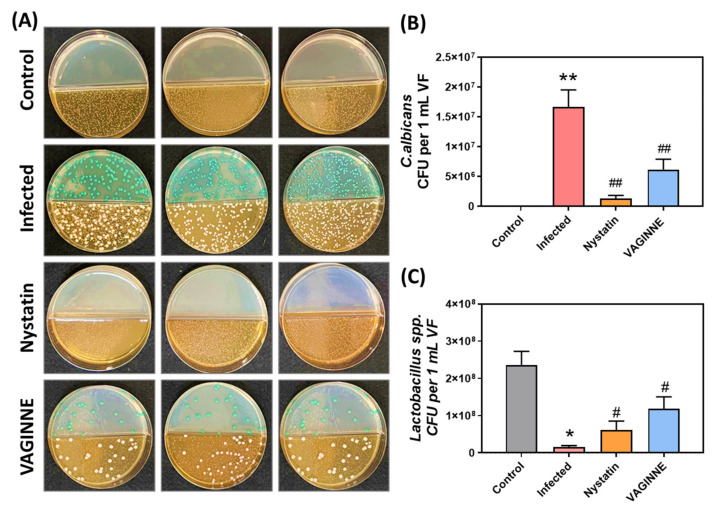
Quantitative assessment of vaginal microbiota following therapeutic intervention. Panel (**A**) demonstrates representative culture plates showing colonization patterns of *C. albicans* (**top**) and *Lactobacillus* species (**bottom**) isolated from vaginal lavage specimens. Graphs display enumeration of (**B**) *C. albicans* and (**C**) *Lactobacillus* populations across treatment cohorts: baseline controls, infection controls (carbomer gel), conventional antifungal treatment (nystatin), and VAGINNE^®^ intervention. Statistical analysis employed one-tailed Mann–Whitney U tests; data expressed as mean ± SEM (*n* = 6). Significant differences indicated as: * *p* < 0.05, ** *p* < 0.01 versus control group; # *p* < 0.05, ## *p* < 0.01 versus infection group. VF: vaginal lavage fluid.

To assess VAGINNE^®^’s efficacy, we analyzed microbial populations in vaginal lavage samples. *Candida* and *Lactobacillus* species were enumerated using selective media (Candida Plus and MRS, respectively). After 48-h culture incubation (30 °C), *Candida* species displayed characteristic chromogenic differentiation on Candida Plus medium: *C. albicans* (green colonies), *C. tropicalis* (blue-gray), *C. glabrata* (lavender-purple), and *C. clostridia* (purple-pink), with other species appearing white. Microscopic examination revealed marked differences in fungal colonization between experimental groups (Figure 3A, Upper panel). The infection control demonstrated elevated *C. albicans* levels (1.67 × 10^7^ CFU/mL), significantly higher than uninfected controls (*p* < 0.01). Both therapeutic interventions effectively reduced fungal burden, with nystatin achieving greater reduction (1.29 × 10^6^ CFU/mL) compared to VAGINNE^®^ treatment (6.15 × 10^6^ CFU/mL). Both treatments showed statistically significant efficacy versus infection controls (*p* < 0.01).

Further examination of *Lactobacillus* spp. growth, shown in Figure 3A (Lower) and Figure 3C, revealed that the Infected group had a significant reduction in CFUs, approximately 1.20 × 10^7^ CFU/mL, compared to the Control group, which had about 2.36 × 10^8^ CFU/mL (*p* < 0.01). However, both the Nystatin (6.18 × 10^7^ CFU/mL) and VAGINNE^®^ (1.19 × 10^8^ CFU/mL) groups showed notable increases in CFUs compared to the Infected group (*p* < 0.05 for both). These findings indicate that treatment with either Nystatin or VAGINNE^®^ effectively reduced *C. albicans* CFUs while promoting the growth of *Lactobacillus* spp. compared to the Infected group treated with carbomer gel. Therefore, VAGINNE^®^ demonstrates significant potential in preserving the vaginal microbiome by enhancing *Lactobacillus* spp. proliferation and concurrently inhibiting *C. albicans* populations. This dual effect underscores the therapeutic promise of VAGINNE^®^ as a candidate for managing VVC, particularly in cases where conventional antifungal treatments are less effective.

### 3.3. Histological Analysis of Vaginal Tissue Following Fermentation Broth Treatment in Infected Mice

**Figure 4:**
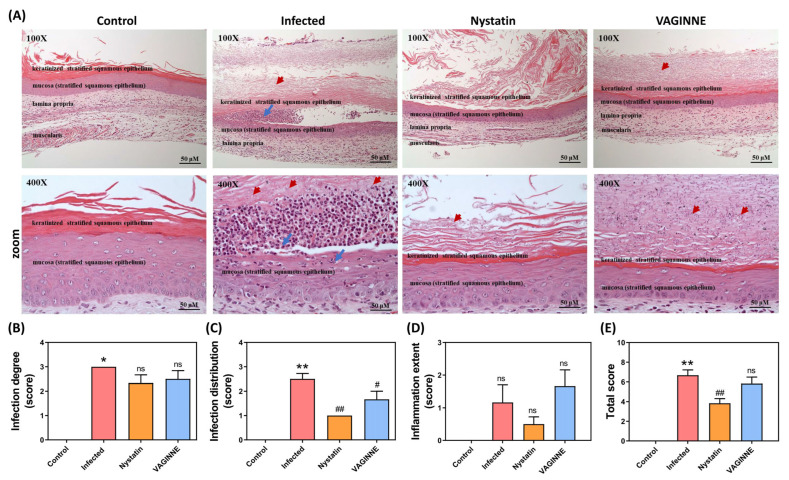
Histological evaluation of vaginal tissues. (**A**) Representative histopathological images of vaginal tissues, alongside scoring for (**B**) infection severity, (**C**) *Candida albicans* distribution, (**D**) extent of inflammation, and (**E**) overall histopathological scores in mice with *C. albicans*-induced infection. Blue arrows indicate the presence of inflammatory cells, while red arrows highlight *C. albicans*. Experimental groups include: Control (untreated mice), Infected (mice treated with carbomer gel), Nystatin (mice treated with nystatin gel), and VAGINNE^®^ (mice treated with VAGINNE^®^ gel). Statistical analyses were conducted using a one-tailed Mann–Whitney U test, with data expressed as mean ± SEM (*n* = 6). Significance is denoted as follows: ns = not significant; * *p* < 0.05 and ** *p* < 0.01 compared to the Control group; # *p* < 0.05 and ## *p* < 0.01 compared to the Infected group. Red arrow: *Candida albicans*; blue arrow: neutrophils.

Histological evaluation of vaginal tissue from uninfected mice revealed a lack of inflammatory cells, indicating a healthy tissue state. In contrast, upon infection with *C. albicans*, a marked increase in inflammatory cell infiltration was observed, underscoring the pathological impact of the infection compared to the controls (Figure 4A). Notably, both VAGINNE^®^ and Nystatin treatments led to a significant decrease in the number of inflammatory cells present in the tissue. In the Infected group, *C. albicans* demonstrated invasive behavior by penetrating keratin layers, leading to notable destruction of the tissue structure (Figure 4A). Conversely, mice receiving treatment with Nystatin or VAGINNE^®^ exhibited only superficial mycelial invasion, which allowed for the preservation of overall tissue integrity. This finding suggests that both treatments effectively limit the depth of *C. albicans* invasion into the keratin, thereby maintaining the vaginal epithelial architecture (Figure 4A).

Furthermore, the vaginal tissues of the infected mice displayed pronounced infection distribution patterns, illustrating the extent of *C. albicans* presence and its impact on local tissue health (Figure 4B,C). Following treatment with VAGINNE^®^, a significant reduction in the extent of *C. albicans* invasion was noted, indicating that VAGINNE^®^ not only alleviates inflammation but also combats the fungal infection effectively. Collectively, these histological findings support the potential of VAGINNE^®^ as a therapeutic agent for restoring tissue health in the context of vaginal candidiasis.

### 3.4. Influence of Fermentation Broth on Hematological Indexes in C. albicans-Infected Mice

Hematological parameters serve as essential biomarkers for assessing inflammatory responses. In our study, the analysis revealed minor fluctuations in monocyte and eosinophil counts among the infected mice compared to the uninfected controls. Specifically, the infected group exhibited elevated levels of these immune cells, indicating an active inflammatory response following *C. albicans* infection. However, after treatment with Nystatin and VAGINNE^®^, both monocyte and eosinophil counts significantly decreased, approaching normal physiological levels (Supplementary Figure S1).

These results suggest that VAGINNE^®^ not only mitigates fungal infection but also aids in restoring the hematological balance disrupted by inflammation. The reduction of these immune cell populations after treatment may reflect a decrease in the overall inflammatory state, providing insight into VAGINNE^®^’s potential as a therapeutic agent for managing inflammatory conditions associated with vaginal candidiasis. This indicates a dual action of VAGINNE^®^ in combating the infection while simultaneously modulating the host’s immune response, thereby contributing to a more balanced inflammatory profile. Further investigations into additional hematological parameters, such as neutrophil and lymphocyte counts, could provide a more comprehensive understanding of VAGINNE^®^’s effects on the immune landscape in the context of VVC.

### 3.5. Fermentation Broth Effects on Proinflammatory Cytokines in Infected Mice

**Figure 5:**
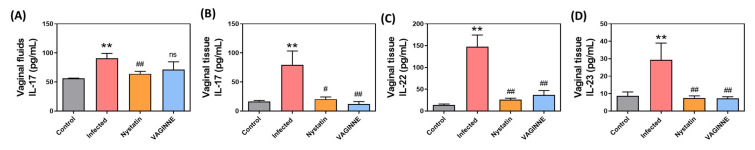
Modulation of pro-inflammatory cytokine levels by VAGINNE^®^ treatment in mice with *Candida albicans* infection. (**A**) IL-17A levels in vaginal lavage fluid and (**B**–**D**) cytokine levels of IL-17A, IL-22, and IL-23 in vaginal tissue were measured across treatment groups. The groups included: Control (untreated mice), Infected (mice treated with carbomer gel), Nystatin (mice treated with nystatin gel), and VAGINNE^®^ (mice treated with VAGINNE^®^ gel). Statistical analyses were conducted using a one-tailed Mann–Whitney U test, with data expressed as mean ± SEM (*n* = 6). Significance levels: ns = not significant; ** *p* < 0.01 compared to the Control group; # *p* < 0.05 and ## *p* < 0.01 compared to the Infected group.

**Figure 6:**
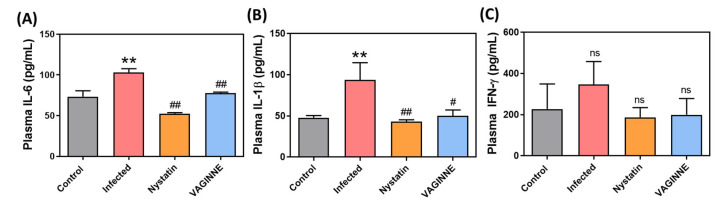
Effects of the fermentation broth from *Lactobacillus crispatus*, *L. gasseri*, and *L. jensenii* on systemic pro-inflammatory cytokines in a murine model of *Candida albicans* vaginal infection. Plasma levels of (**A**) IL-6, (**B**) IL-1β, and (**C**) IFN-γ were assessed among four groups: Control (untreated mice), Infected (carbomer gel-treated mice), Nystatin (nystatin gel-treated mice), and VAGINNE^®^ (VAGINNE^®^ gel-treated mice). Cytokine levels were measured to evaluate the anti-inflammatory effects of the treatments. Statistical analysis was conducted using a one-tailed Mann–Whitney U test, with data presented as mean ± SEM (*n* = 6). Significance levels: ns = not significant; ** *p* < 0.01 compared to the Control group; # *p* < 0.05 and ## *p* < 0.01 compared to the Infected group.

Proinflammatory cytokine levels were assessed in the vaginal lavage fluid, vaginal tissue, and plasma of the mice, as shown in Figure 5 and Figure 6. Infected mice exhibited significantly elevated levels of IL-17A in both vaginal lavage fluid and tissue, along with increased levels of IL-22 and IL-23 in the vaginal tissue, and heightened concentrations of IL-6 and IL-1β in plasma. There was also a noted upward trend in plasma IFN-γ levels. Post-treatment with VAGINNE^®^, there was a significant reduction in IL-17A, IL-22, and IL-23 in vaginal tissue, as well as decreased IL-6 and IL-1β levels in plasma. Additionally, levels of IL-17A in vaginal lavage fluid and IFN-γ in plasma showed a declining trend. These results indicate that *C. albicans* infection enhances proinflammatory cytokine levels, while VAGINNE^®^ treatment effectively mitigates these inflammatory markers, highlighting its potential therapeutic benefits in managing inflammatory responses associated with vaginal candidiasis.

## 4. Discussion

*Lactobacillus* species are the predominant microorganisms in the healthy human vaginal microbiome, with *L. crispatus*, *L. gasseri*, *L. iners*, and *L. jensenii* being the most commonly isolated strains [14,15]. These bacteria are characterized by their gram-positive nature, microaerophilic metabolism, acid tolerance, inability to form spores, and capacity to produce lactic acid [27]. These characteristics contribute to their ability to maintain a healthy vaginal environment and protect against pathogenic microorganisms.

VAGINNE^®^, the subject of this study, is a novel formulation derived from the fermentation broth of *L. crispatus, L. gasseri*, and *L. jensenii.* The selection of these specific *Lactobacillus* species is based on their demonstrated antagonistic effects against *Candida albicans* and other vaginal pathogens. In vitro studies have shown that supernatants from cultures of these *Lactobacillus* species can significantly reduce the filamentation and growth of *C. albicans* [17]. This inhibitory effect is attributed to various mechanisms, including the production of antimicrobial compounds, competition for nutrients and adhesion sites, and modulation of the host immune response. The efficacy of *Lactobacillus* species in managing vaginal infections has been demonstrated in animal models. For instance, Li et al. [28] reported that *L. crispatus* could inhibit 60 to 70% of *C. albicans* growth in a rat model of VVC compared to untreated controls. This significant reduction highlights the potential of *Lactobacillus*-based therapies in managing VVC. The protective effects of *L. crispatus* against sexually transmitted infections (STIs), bacterial vaginosis (BV), and VVC have been extensively studied [29,30]. These effects are primarily attributed to its ability to produce lactic acid and bacteriocins. Lactic acid production is essential for maintaining the acidic pH of the vaginal environment, creating conditions that are inhospitable to many pathogens. Furthermore, lactic acid may inhibit the hyphal growth and adhesion of *C. albicans*, providing additional protection against infections. Bacteriocins, on the other hand, are antimicrobial peptides that can directly inhibit the growth of competing microorganisms.

Furthermore, the cell-free supernatant derived from *L. crispatus* cultures has demonstrated superior efficacy in inhibiting *Candida* colonization compared to that of *L. iners* [31]. This enhanced activity is indicative of a higher concentration of protonated lactic acid [32]. The protonated form of lactic acid is particularly effective in penetrating microbial cell membranes, disrupting intracellular pH, and inhibiting metabolic functions of pathogens [16]. The combination of *L. crispatus*, *L. gasseri*, and *L. jensenii* in VAGINNE^®^ aims to harness the complementary protective mechanisms of these *Lactobacillus* species. While *L. crispatus* is known for its strong lactic acid production, *L. gasseri* has been associated with the production of hydrogen peroxide, another antimicrobial compound. *L. jensenii*, on the other hand, has been shown to produce unique bacteriocins that can inhibit a wide range of pathogens.

By utilizing the fermentation broth of these three *Lactobacillus* species, VAGINNE^®^ potentially offers a multi-faceted approach to managing VVC. The fermentation process allows for the accumulation of various metabolites and antimicrobial compounds produced by the *Lactobacillus* strains, which may act synergistically to inhibit *C. albicans* growth and virulence. This comprehensive approach, leveraging the natural defense mechanisms of vaginal *Lactobacillus* species, represents a promising alternative to conventional antifungal treatments for VVC. It aligns with the growing interest in microbiome-based therapies and may offer advantages in terms of reduced side effects and a lower risk of developing antifungal resistance.

Currently, there are no established in vivo assays to assess the anti-Candida effects of fermentation broth derived from *Lactobacillus crispatus*, *L. gasseri*, and *L. jensenii*. In this study, we evaluated the therapeutic efficacy of VAGINNE^®^ in a mouse model of VVC induced by *C. albicans* infection in BALB/c mice. Treatment with VAGINNE^®^ resulted in significant reductions in the proliferation of *C. albicans* and alleviated inflammatory infiltration in vaginal tissues, similar to the effects observed with nystatin. Although the treatment led to an approximate tenfold reduction in *Candida* cell counts, complete recovery of the mice and prevention of reinfection could not be guaranteed. Nonetheless, VAGINNE^®^ demonstrated protective effects on the vaginal microbiome by promoting the growth of *Lactobacillus* species while concurrently suppressing *C. albicans* populations. We hypothesize that the observed anti-Candida activity of VAGINNE^®^ may be partially attributed to its content of bacteriocins, hydrogen peroxide, and lactic acid [17]. Failure to restore healthy flora, particularly *Lactobacillus*, may contribute to the recurrence of VVC following standard treatments. However, our study indicates that VAGINNE^®^ treatment can effectively restore *Lactobacillus* populations, potentially reducing the risk of recurrent VVC (RVVC). *Lactobacillus*, as a beneficial probiotic, plays a critical role in the health of individuals experiencing VVC [32]. By producing lactic acid, *Lactobacillus* exhibits antibacterial, antiviral, and immunomodulatory properties, which is essential for inhibiting *C. albicans* virulence [33]. Moreover, *Lactobacillus* species generate various metabolites and bacteriocins that further suppress pathogenic growth [34,35]. Notably, *L. gasseri* has been identified to produce 1-acetyl-β-carboline, while *L. jensenii* and *L. crispatus* may secrete antimicrobial peptides and proteins [17,36]. Collectively, the therapeutic effects of VAGINNE^®^ in this study underscore its potential role in preserving vaginal microbiome integrity and combating *C. albicans* infections [37,38].

In our study, we found that VAGINNE^®^ significantly protected vaginal tissues by reducing inflammatory cell infiltration and limiting the spread of *Candida albicans* infection in mice. The treatment with VAGINNE^®^ not only alleviated clinical signs of vaginal inflammation but also led to a notable suppression of pro-inflammatory cytokines. Specifically, levels of IL-1β and IL-6 in plasma, as well as IL-17A, IL-22, and IL-23 in vaginal tissues, were markedly decreased in VAGINNE^®^-treated mice. These cytokines are crucial for the proliferation and differentiation of immune cells; IL-1β and IL-6 facilitate immune responses, while IL-23 activates naive CD4+ T cells, promoting their differentiation into Th17 cells, which then produce IL-17 and IL-22. These latter cytokines stimulate vaginal epithelial cells to secrete antimicrobial peptides, thereby enhancing local defenses against *C. albicans* and contributing to overall vaginal health [37,38].

The anti-inflammatory effects of VAGINNE^®^ play a crucial role in its protective action against VVC, likely attributable to its lactic acid content. Lactic acid possesses well-documented immunomodulatory properties, which significantly suppress the production of inflammatory cytokines by epithelial cells. This reduction in cytokine levels can effectively attenuate the overall inflammatory response, thereby mitigating the symptoms associated with VVC. By enhancing the vaginal environment’s acidity and inhibiting pro-inflammatory signaling, VAGINNE^®^ may promote a more favorable condition for maintaining vaginal health and combating infections [39,40].

A key constraint in our experimental design was the restricted analysis of inflammatory markers across different sample types. Due to the finite biological samples obtainable from each mouse, we prioritized IL-17A measurements in both vaginal secretions and tissue specimens, while restricting the analysis of IL-22 and IL-23 to tissue samples only. This focused sampling strategy aligned with animal welfare guidelines by minimizing the number of experimental subjects. The decision to emphasize IL-17A analysis across multiple sample types was based on its established role as a crucial inflammatory mediator in mucosal immunity. However, this selective approach meant we could not fully characterize the distribution patterns of other cytokines between tissue compartments and biological fluids. A more comprehensive analysis might have revealed important insights about the temporal and spatial dynamics of IL-22 and IL-23 during the inflammatory response.

This study highlights the anti-Candida and anti-inflammatory potential of VAGINNE^®^ using a BALB/c mouse model, but its applicability to humans remains limited. Mouse models, despite their utility in preclinical research, differ significantly from humans in vaginal microbiota composition, immune responses, and disease progression. These discrepancies may influence the translational relevance of the findings. Therefore, further clinical trials in human populations are essential to confirm the safety, efficacy, and mechanisms of VAGINNE^®^ in treating vulvovaginal candidiasis effectively.

## 5. Conclusions

The fermentation broth from *Lactobacillus crispatus*, *Lactobacillus gasseri*, and *Lactobacillus jensenii* exhibited significant inhibitory effects on *Candida albicans* infection and associated inflammatory responses in a mouse model of VVC. The notable decrease in *C. albicans* levels in vaginal lavage, alongside reduced infection and inflammation, underscores its therapeutic potential. Additionally, the broth’s positive impact on promoting *Lactobacillus* growth indicates its role in maintaining a healthy vaginal microbiome. These findings position the fermentation broth as a promising anti-Candida and anti-inflammatory treatment option for VVC.

## Data Availability

The original contributions presented in the study are included in the article/Supplementary Materials, further inquiries can be directed to the corresponding authors.

## Supplementary Materials

The following supporting information can be downloaded at: https://www.mdpi.com/article/10.3390/jof11010018/s1, Figure S1: Effects of the fermentation broth derived from *Lactobacillus crispatus*, *L. gasseri*, and *L. jensenii* on hematological indexes, including (A) neutrophils, (B) lymphocytes, (C) monocytes, (D) eosinophils, and (E) basophils in mice with *Candida albicans* vaginal infection. Significant differences indicated as: ns = not significant, * *p* < 0.05 versus control group; # *p* < 0.05, versus infection group. Table S1: Scoring criteria for infection degree of vaginal tissue. Table S2: Scoring criteria for infection distribution of vaginal tissue.
