# Supplementary material for: Dual Mechanisms of Action: Anti-Candida and Anti-Inflammatory Potential of Lactobacillus Fermentation Broth in Treating Vulvovaginal Candidiasis
Source: J Fungi (Basel). 2024 Dec 30;11(1):18. doi: 10.3390/jof11010018 (PMC11766182; doi:10.3390/jof11010018)
Supplement: Supplementary file 1 [file jof-11-00018-s001.zip › jof-3271738-supplementary.pdf]

Supplementary data

Supplementary Table S1. Scoring criteria for infection degree of vaginal tissue

Score	Infection degree	Grading
0	Absent	No yeast elements
1	Mild	Meaning 1-30 yeast elements in the vaginal lumen and cornified epithelial layer, or ≥ 5 in the upper 1/3 layer of the vaginal mucosa
2	Moderate	Meaning 31-60 yeast elements in the vaginal lumen and cornified epithelial layer or ≥ 5 in the upper 2/3 layer of the vaginal mucosa
3	Severe	Meaning >60 yeast elements in the vaginal lumen and cornified epithelial layer or ≥ 5 in the whole layer of the vaginal mucosa

Supplementary Table S2. Scoring criteria for infection distribution of vaginal tissue

Score	Infection distribution
0	Normal
1	1-33% of the affected
2	34-66% of the affected
3	67-100% of the affected

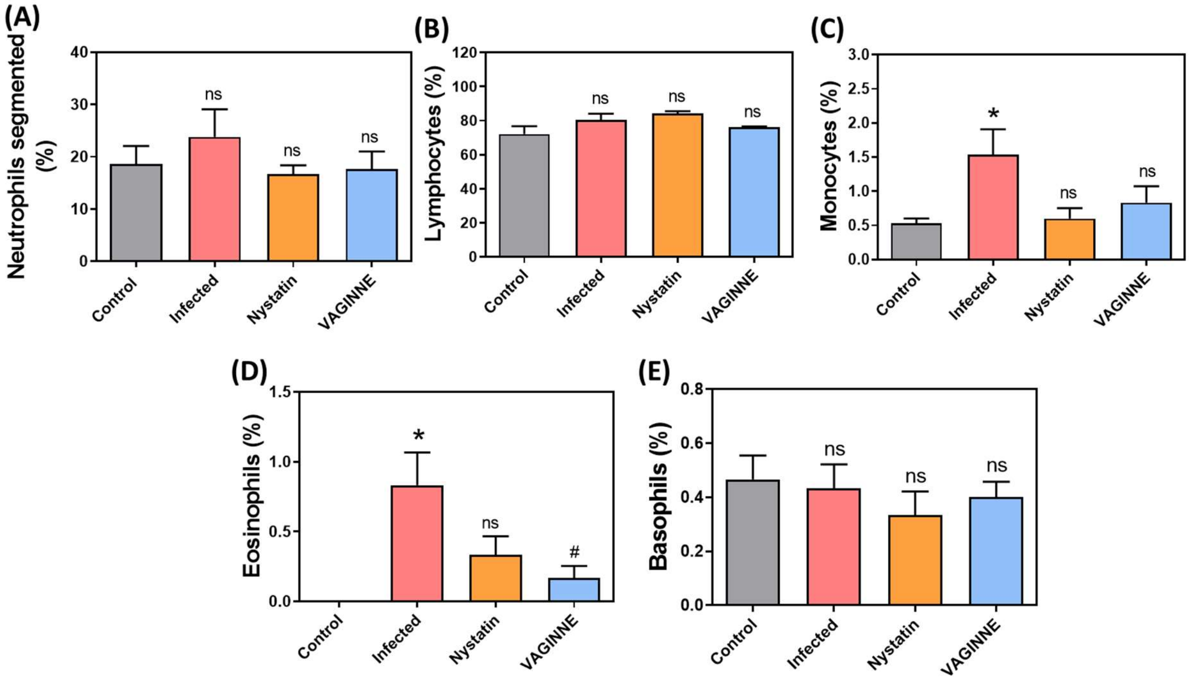

Supplementary Figure S1. Effects of the fermentation broth derived from *Lactobacillus crispatus*, *L. gasseri*, and *L. jensenii* on hematological indexes, including (A) neutrophils, (B) lymphocytes, (C) monocytes, (D) eosinophils, and (E) basophils in mice with *Candida albicans* vaginal infection. Significant differences indicated as: ns = not significant, * $p < 0.05$ versus control group; # $p < 0.05$, versus infection group.
